# Locating hyperfunctioning parathyroid glands using ^11^C-Choline PET/CT: an inter- and intra-observer variation study

**DOI:** 10.1186/s41824-021-00108-z

**Published:** 2021-07-06

**Authors:** Julie Wulf Christensen, Lars Thorbjørn Jensen, Susanne Bonnichsen Søndergaard, Rikke Broholm, Christian Haarmark, Martin Krakauer, Finn Noe Bennedbæk, Bo Zerahn, Waldemar Trolle, Christoffer Holst Hahn, Bent Kristensen

**Affiliations:** 1 Department of Nuclear Medicine, Herlev and Gentofte Hospital, 2730 Herlev, Denmark; 2 Department of Clinical Physiology and Nuclear Medicine, Bispebjerg and Frederiksberg Hospital, 2400 Copenhagen, Denmark; 3 Department of Medicine, Division of Endocrinology, Herlev and Gentofte Hospital, 2730 Herlev, Denmark; 4 Department of Otorhinolaryngology and Neck Surgery, North Zealand Hospital, 3400 Hilleroed, Denmark; 5 Department of Otorhinolaryngology and Neck Surgery, Rigshospitalet, 2100 Copenhagen, Denmark

**Keywords:** ^11^C-Choline PET/CT, Hyperparathyroidism, Inter-observer agreement, Intra-observer agreement

## Abstract

**Background:**

Use of ^11^C-Choline PET/CT is gaining ground in detecting hyperfunctioning parathyroid glands in primary hyperparathyroidism. The purpose of this study was to evaluate the robustness of ^11^C-Choline PET/CT by assessing intra- and inter-observer agreement to determine whether the method was reader sensitive and therefore should only be performed at highly specialised sites with a high number of cases.

PET/CT images of 40 patients diagnosed with primary hyperparathyroidism were anonymised and evaluated three times by three readers: an expert reader and two non-experts (non-experts were experienced in PET/CT imaging, but not in ^11^C-Choline PET/CT in the setting of primary hyperparathyroidism). Number of hyperfunctioning parathyroid glands, location relative to the thyroid gland and confidence of each assessment (low, moderate or high) were noted, and intra- and inter-observer agreement calculated using Fleiss’ kappa method. Sensitivities and specificities of the non-experts were calculated using the expert reader as gold standard.

**Results:**

Intra-observer agreement was ‘good’ to ‘near perfect’ for all readers. Inter-observer agreement was good between non-experts and the expert, with kappa values ≥ 0.74. Sensitivities between non-experts and the expert were high, > 81%, when assessing which side and 75% when assessing thyroid quadrant. All specificities were > 94%. Reader certainties were ‘high’ in > 80% of cases for the expert and > 70% and > 65%, respectively for the two non-experts.

**Conclusion:**

^11^C-Choline PET/CT is not reader sensitive for the localisation of hyperfunctioning parathyroid glands and may therefore be safely implemented at sites that have a moderate number of cases. Access to a cyclotron laboratory is, however, a necessity for the production of ^11^C-Choline.

The study was conducted in accordance with the Helsinki 2 declaration and The International Council for Harmonisation Guideline for Good Clinical Practice (ICH_GCP) clinical trial, approved by the Research Ethics Committee of the Capital Region of Denmark (Journal-nr.:H-18012490, date of approval: 18 June 2018) and the Danish Medicines Agency (EudraCT no. 2018-000726-63, date of approval: 6 June 2018). The GCP unit in Eastern Denmark has carried out regular monitoring of the trial according to GCP (ID: 2018-1050).

**Supplementary Information:**

The online version contains supplementary material available at 10.1186/s41824-021-00108-z.

## Background

Primary hyperparathyroidism (PHPT) is a disease caused by hyperfunctioning tissue in one or more of the parathyroid glands. The incidence in Denmark is 600–700 per year or 100 per million citizens (Danish Endocrine Society 2021). The standard curative treatment of PHPT is parathyroidectomy (PTx), the surgical removal of the hyperfunctioning parathyroid gland(s) (HPGs). In order to offer minimally invasive surgery, PTx is preceded by localisation imaging, where the precise location of the HPG(s) is determined (Danish Endocrine Society 2021; Rolighed et al. 2014).

Several imaging modalities are available, including newer methods such as ^11^C-Choline positron emission tomography/computed tomography (^11^C-Choline PET/CT). The benefits of the ^11^C-Choline PET/CT are low effective radiation dose (6.6 millisievert) to patients and staff, its simple preparation and short acquisition time leading to a higher capacity as compared to ^99m^Tc-MIBI/^123^iodide subtraction single-photon emission computed tomography/computed tomography (SPECT/CT) and planar pinhole imaging (henceforth, referred to as Di-SPECT) (≈13 millisievert). In a prospective cohort study of 60 patients diagnosed with PHPT, we have recently reported a sensitivity for locating HPGs of 1.00 (95% CI, 0.93–1.00) at the patient level and 0.87 (95% CI, 0.76–0.93) at the gland level (Ismail et al. 2020).

Choline PET/CT is gradually replacing conventional imaging in many centres but is still considered a relatively new method, with fewer than 1000 scans reported in studies worldwide. Consequently, true nuclear medicine experts are few and knowledge is lacking on the amount of training needed before an expert level is reached. Thus, it is as yet uncertain whether ^11^C-Choline PET/CT imaging of PHPT patients should be restricted to highly specialised centres. If the method is robust and shows good inter- and intra-observer agreement, it would be feasible to disseminate the method to centres with fewer annual cases and, by extension, less experience. If dissemination were possible, the method would be accessible to more patients. However, this cannot come at the cost of lower quality, which is why knowledge of inter- and intra-observer agreement is beneficial. With a short half-life of only 20 min, ^11^C-Choline must be produced on-site, and the ^11^C-Choline PET/CT method can only be implemented at centres with access to a cyclotron laboratory. ^18^F-Choline is a similar tracer, which with its longer half-life may be easier to disseminate, but whether the two tracers are interchangeable has to our knowledge, not yet been confirmed.

Noltes et al. have examined the inter-observer agreement between four observers for four separate scam durations and found moderate-good kappa values (Fleiss kappa between 0.553 and 0.587) (Noltes et al. 2019).

In our recent paper (Ismail et al. 2020), all images were analysed by an expert reader with a sensitivity of 87% (95% CI, 76–93%) with the surgeon’s report as a reference. The purpose of the present study is to assess how accurately readers with varying expertise can replicate their interpretations of the images (i.e. intra-observer variation) as well as how accurately and confidently non-expert readers can assess the same images (i.e. inter-observer variation). The robustness of the method will be indicative of whether it can be taken into routine use by facilities with a moderate number of scans per year (i.e. approx. 100-150 yearly scans) or should be limited to a few highly specialised centres.

To our knowledge, no further investigation of inter- or intra-observer agreement of ^11^C-Choline or the alternative ^18^F-Choline has been made.

## Methods

For the study described in our previous paper, we prospectively included 60 patients from August 2018 to May 2019 (Ismail et al. 2020). All patients were diagnosed with PHPT and referred to preoperative location imaging using Di-SPECT and ^11^C-Choline PET/CT, and later to PTx. Of these, the 40 consecutive patients to first complete both imaging modalities and PTx were included in the present study. PET/CT images were re-anonymised and reassessed in order to determine inter- and intra-observer variation. Only the ^11^C-Choline PET/CT images were re-evaluated in this paper.

All 40 PET/CT images were given three new anonymised IDs in three randomised orders and thus evaluated three times. Each PET/CT image was evaluated by three readers, all blinded to the established truth (as determined by surgery and postoperative pathology and biochemistry) as well as to each other’s and their own previous results.

The three readers were all nuclear medicine specialists:

1. Expert reader: Highly experienced in PET/CT imaging and as well as parathyroid imaging in general including experience in ^11^C-Choline PET/CT. At the time the study began, the expert reader had evaluated approx. 70 parathyroid ^11^C-Choline PET/CT studies and had more than 20 years of nuclear medicine experience.
2. Non-expert 1: Experienced in PET/CT imaging (primarily oncology) and some experience with parathyroid imaging. No experience with ^11^C-Choline PET/CT. Eight years of nuclear medicine experience.
3. Non-expert 2: Experienced in PET/CT imaging (primarily oncology) and sparse experience with parathyroid imaging. Some experience with ^11^C-Choline PET/CT for detection of prostate cancer metastases. Ten years of nuclear medicine experience.

Readers were given a coded sheet and asked to assess (a) number of HPGs, (b) which side (right, left or both), (c) location relative to the thyroid gland (upper, middle, lower third or ectopic) and (d) confidence of response (low, moderate or high). All 40 images were assessed three times by each reader. Analyses were performed using the MIM software (MIM Software Inc., USA).

Assessments (b) and (c) leave it to the observer to choose between seven possible locations. Differentiation between the upper and middle third of the thyroid gland is notoriously difficult and imprecise. Therefore, we combined the upper and middle third into one location, and each ectopic location with the appropriate standard gland location. The result was a total of four possible locations, concurrent with normal anatomy. In order to simplify statistics, we assumed a total of four parathyroid glands per patient.

We have not taken into consideration the ‘true’ gland pathology as determined by surgery because our focus was on the reproducibility of image evaluation. For results on the former, we refer to our publication on this subject (Ismail et al. 2020).

### Statistical analyses

Statistical analyses were conducted using ‘R’ version 4.0.2 (R Core Team, 2020). R: A language and environment for statistical computing. R Foundation for Statistical Computing, Vienna, Austria (URL https://www.R-project.org/) and the two additional packages irr: Various Coefficients of Interrater Reliability and Agreement. R package version 0.84.1 and vcd: Visualizing Categorical Data. R package version 1.4-8 (Meyer et al. 2020; Gamer et al. 2019).

Intra-observer agreement was assessed using Fleiss’ kappa method, comparing all three rounds for each reader using four possible parathyroid locations (i.e. upper right, lower right, upper left and lower left). Kappa values were interpreted as follows: < 0.00, poor; 0.00–0.20, slight; 0.21–0.40, fair; 0.41–0.60, moderate; 0.61–0.80, good and > 0.80, almost perfect agreement (Landis and Koch 1977).

In order to avoid possibly assessing on a learning curve, the third and final round of image analysis from each reader was used to assess inter-observer agreement. Fleiss’ kappa was used in the same manner as described for inter-observer agreement to compare each of the two non-experts to the expert reader. We then calculated sensitivities and specificities of each of the non-experts, using the expert reader as the gold standard. All patients were diagnosed with PHPT and, as such, analysis at the patient level (i.e. HPG/no HPG) is irrelevant. Rather, sensitivities and specificities were calculated with regard to the side of the thyroid gland (left/right, *N* = 80) and location in relation to the thyroid (upper left, upper right, lower left or lower right, *N* = 160), whilst adjusting for clustered observations (i.e. multiple observations per patient) using a so-called ‘sandwich estimator’ of variance with correlation adjusted confidence intervals (Pustejovsky 2020; Gopstein 2018; Genders et al. 2012; Obuchowski 1998). ‘True positives’ and ‘true negatives’ were defined as cases in which the non-expert made the same assessment as the expert.

To visualise the degree of both inter- and intra-observer agreement and disagreement, we created Bangdiwala’s Observer Agreement Charts (Bangdiwala 2017). Unlike a single estimate given by the kappa analysis, the agreement chart allows a visual estimate of possible bias amongst observers via comparison of row and column totals (marginal totals) from the contingency table and shown as grey-scaled/shaded rectangles. In cases of perfect agreement, the rectangles are depicted as black squares lying exactly on the diagonal line. Disagreements are shown as white rectangles. Bias is assessed by the deviation of the rectangles away from the red diagonal line. The further away the ‘path’ of the rectangles is from the diagonal line, the larger the possible bias. For all reported confidence intervals, a level of 95% was chosen, and all reported *p* values are exact. Average confidence scores were calculated as the average (where low confidence = 1, moderate confidence = 2 and high confidence = 3).

## Results

^11^C-Choline PET/CT images from 40 patients were consecutively included for reanalysis. For inclusion criteria and thorough imaging protocol, please refer to the paper by Ismail et al. (Ismail et al. 2020). Patients had a median age of 61 (range, 34–83 years) and were predominantly women (78%). Two patients had previously undergone PTx; in one case, a single HPG was removed and in the other no prior HPG was located. Readers were not given this information. See Table 1 for patient characteristics. See the supplementary material (Table S1) for number and location of HPGs as described by each reader. Table 2 displays the confidence scores of each reader in each round. There was a high number of high confidence scores from the expert reader (≥ 80%), whilst the ratio of high confidence scores was slightly lower from the non-experts (65–75%). The calculated average confidence score ranged from 2.6 to 2.9.

**Table 1:** Baseline patient characteristics

		N	(%)
Number of patients, *n*		40	
Gender	Female	31	(78%)
	Male	9	(23%)
Previous surgery	Yes	2^a^	(5%)
	No	38	(95%)
	**Median**	**Range**	
Age	61	34–83	
Height (*cm*)	170	153–191	
Weight (*kg*)	77	58–119	
BMI (*kg/m*^*2*^)	26.7	20.3–41.7	
Preoperative Ca^2+^ (*mmol/L)*^b^	1.48	1.33–1.76	
Preoperative PTH (*pmol/L*)^c^	14.8	10.0–53.5	

^a^A single HPG was removed in total

^b^Plasma ionised calcium; reference value, 1.18–1.32

^c^Plasma parathyroid hormone; reference value, 2.0–8.5

**Table 2:** Confidence scores by round and reader

	Confidence	Round 1		Round 2		Round 3	
		N	(%)	N	(%)	N	(%)
**Expert reader**	Low	3	(7.5)	1	(2.5)	5	(12.5)
	Moderate	5	(12.5)	4	(10.0)	2	(5.0)
	High	32	(80.0)	35	(87.5)	33	(82.5)
	Average^a^	2.7		2.9		2.7	
**Non-expert 1**	Low	4	(10.0)	5	(12.5)	2	(5.1)
	Moderate	8	(20.0)	7	(17.5)	9	(23.1)
	High	28	(70.0)	28	(70.0)	28	(71.8)
	Average^a^	2.6		2.6		2.7	
**Non-expert 2**	Low	6	(15.0)	4	(10.0)	3	(7. 5)
	Moderate	7	(17.5)	10	(25.0)	7	(17.5)
	High	27	(67.5)	26	(65.0)	30	(75.0)
	Average^a^	2.5		2.6		2.7	

^a^Calculated as the average of certainties, where low = 1, moderate = 2 and high = 3

**Fig. 1:**
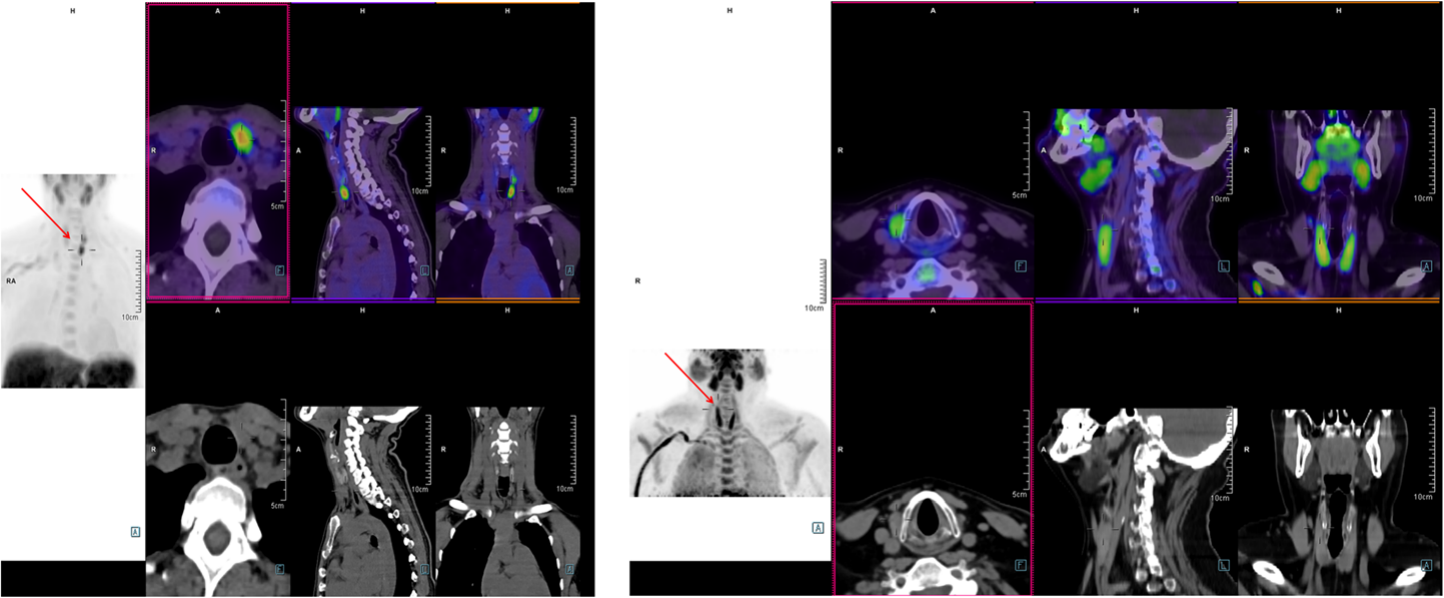
Two very different ^11^C-Choline PET/CT images. ^11^C-Choline positron emission tomography/computed tomography (PET/CT). Arrows mark the suspected hyperfunctioning parathyroid gland. Left: Image deemed of ‘high certainty’ by all three readers. Right: Image deemed of ‘low certainty’ by the expert reader (no images were deemed of low quality by all three readers). In the image to the right, choline uptake in the thyroid and salivary glands is clearly seen

PET/CT images vary in clarity. Figure 1 displays the variation in image clarity, with an image deemed ‘high Confidence’ by all 3 readers alongside one deemed ‘low confidence’ by the expert.

### Intra-observer agreement

As the variance was minimal, calculating kappa for number of HPGs was not possible and the results for ‘number of HPGs’ were therefore omitted from the following. Kappa values and 95% confidence intervals for intra-observer agreement for each reader are displayed in Table 3. All kappa values are high: for the expert and non-expert 1 readers, they are consistently above 0.8 (near perfect agreement), whilst for non-expert 2 intra-observer agreement borders between ‘good’ and ‘near perfect’ agreement according to our chosen scale of 4.

**Table 3:** Kappa values for intra-observer agreement

	Expert		Non-expert 1		Non-expert 2	
	Kappa	(95% CI)	Kappa	(95% CI)	Kappa	(95% CI)
Left/right	0.90	(0.82-0.97)	0.90	(0.82-0.98)	0.78	(0.68-0.88)
Location (1-4)	0.87	(0.80-0.93)	0.91	(0.84-0.97)	0.81	(0.73-0.89)

**Fig. 2:**
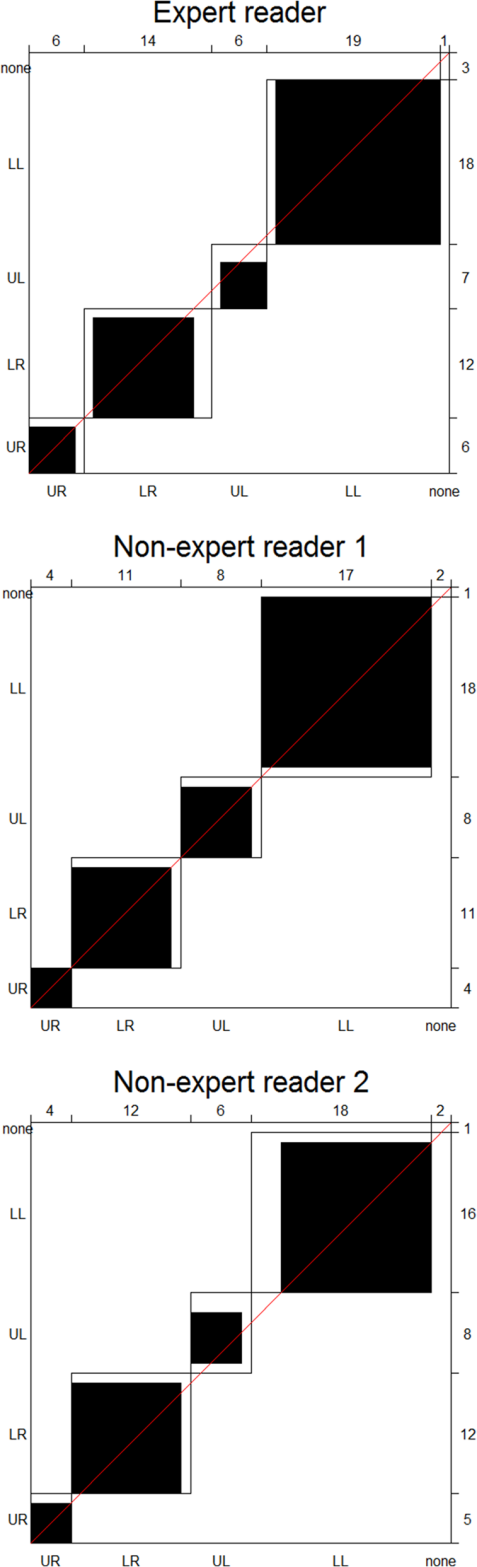
Agreement plots for intra-observer agreement on anatomical location. From the expert, non-expert 1 and non-expert 2 readers. UR, upper right; LR, lower right; UL, upper left; LL, lower left. Due to the high number of none-none agreements these have been omitted for the agreement plots, but can be seen in the supplementary material

Figure 2 displays the agreement plots for all three readers individually. In all three cases, the black squares lie on the diagonal line with only minor white areas, suggesting no bias in accordance with the high kappa values. The majority of HPGs are located in the lower left and lower right quadrant in accordance with findings by Borumandi et al. as well as Lavely et al. (Borumandi et al. 2019; Lavely et al. 2007). The number of none-none agreement (i.e. where both readers find no HPG) was high (111, 118 and 113 from the expert, non-expert 1 and non-expert 2, respectively) and would overwhelm the agreement plots. For that reason, they have been omitted but are available in the supplementary material (Figure S1). The ‘none-disagreements’, (i.e. where one reading finds an HPG in the upper left quadrant but another reading finds nothing) are included in the plots and marked by a white rectangle in the upper right corner of the plot. These cases are few, indicating that it is rare for the same observer to miss an HPG on one reading and identify it the next.

### Inter-observer variation

Tables 4 and 5 show kappa values as well as sensitivities and specificities respectively at both patient level and gland level when using the expert reader as the gold standard.

**Table 4:** Kappa values for inter-observer agreement between the expert and non-expert readers

	Expert – non-expert 1		Expert – non-expert 2		Non-expert 1 – Non-expert 2	
	Kappa	(95% CI)	Kappa	(95% CI)	Kappa	(95% CI)
Left/right	0.75	(0.60–0.88)	0.80	(0.65–0.92)	0.85	(0.73–0.95)
Location^a^	0.74	(0.62–0.85)	0.78	(0.65–0.88)	0.83	(0.72–0.91)

^a^Location relative to the thyroid gland, i.e. upper left, upper right, lower left or lower right

**Table 5:** Sensitivity and specificity of non-experts, using the expert as the gold standard

	Non-expert 1				Non-expert 2			
	Sensitivity	(95% CI)	Specificity	(95% CI)	Sensitivity	(95% CI)	Specificity	(95% CI)
Left/right	82	(66–91)	94	(88–98)	86	(72–94)	94	(87–98)
Location^a^	76	(54–89)	96	(89–98)	80	(57–93)	96	(88–99)

^a^Location relative to the thyroid gland, i.e. upper left, upper right, lower left or lower right

Though not quite as high as intra-observer agreement, kappa values for inter-observer agreement were still above 0.74 and thus at the high end of ‘good’ agreement. When assessing side of the thyroid, specificities were high at 94% from both non-experts, and sensitivities only slightly lower at 82% (95% CI, 66–91%) and 86% (95% CI, 72–94%) respectively. With regards to location, findings were similar, with specificities of 96% from both non-experts and sensitivities at 76% (95% CI, 63–85%) and 80% (95% CI, 69–88%).

**Fig. 3:**
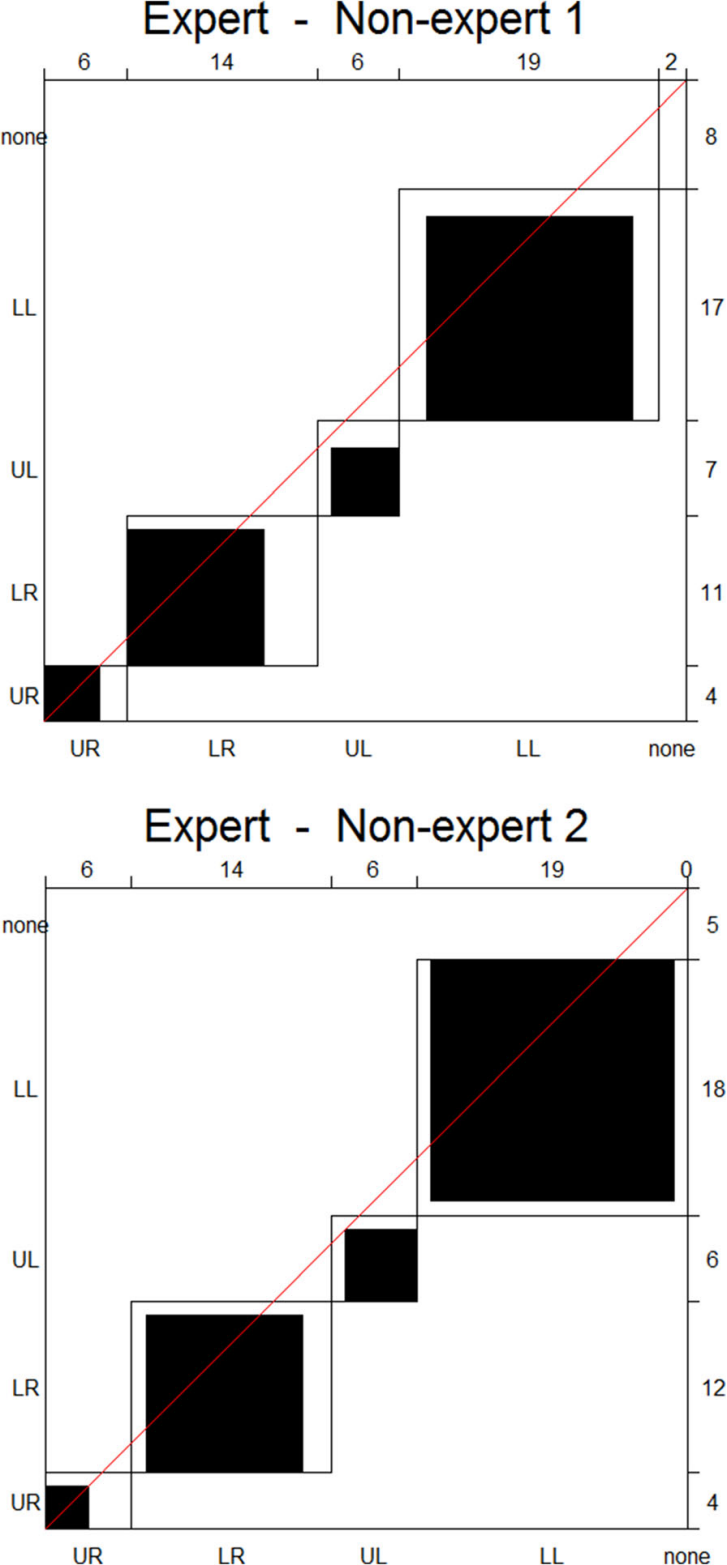
Agreement plots for inter-observer agreement on anatomical location. From the expert reader compared to the two non-experts individually. Left, non-expert 1; right, non-expert 2. The expert is marked on the x-axis and the non-expert on the y-axis. UR, upper right; LR, lower right; UL, upper left; LL, lower left. Due to the high number of none-none agreements, these have been omitted for the agreement plots, but can be seen in the supplementary material

In the inter-observer agreement plots displayed in Fig. 3, the black squares are relatively large and are of the same order of magnitude as those of the intra-observer comparisons. However, both comparisons have a slight tendency to lie below the diagonal line, suggesting a minor negative bias for one of the readers in both plots as indicated by a slight decrease in the kappa values. There is a higher number of ‘none-disagreements’ (shown in the upper right corner) than in the intra-observer plots. This indicates that in eight and five cases respectively the expert finds HPGs that the non-experts do not. This does not necessarily imply that they find nothing in that particular image, but rather that the expert finds more (i.e. the expert finds HPGs in the upper left and lower right quadrant, whilst the non-expert merely finds one in the upper left). The ‘none-none agreements’ (110 in both cases) have been omitted from the agreement plots due to their high number but can be seen in the supplementary material (Figure S2).

## Discussion

^11^C-Choline PET/CT is gradually gaining ground as a reliable method of localising HPGs preoperatively, which due to its shorter preparation, shorter scan time and lower radiation dose, has advantages over Di-SPECT. In expert hands, the method has proven to be highly accurate and is a likely candidate to be the new routine method wherever possible. Before this can happen, it is important to assess whether nuclear medicine specialists are able to assess the images reliably and with high agreement intra- and inter-individually as is the case with Di-SPECT (Lavely et al. 2007; Tunninen et al. 2013).

Intra-observer agreement was found to be high for the identification and localisation of HPGs when using ^11^C-Choline PET/CT in patients with primary hyperparathyroidism. Although slightly lower in one non-expert, the kappa value was consistently above 0.78. For this reader, there was a slight increase in confidence from round 1 to round 3, which could be interpreted as learning with a subsequent confidence increase from experience. This indicates that perhaps a learning curve of approximately 40 images is prudent. In both expert and non-expert 1, there is a near perfect kappa score and no clear pattern in the confidence scores. Agreement was slightly higher for location left or right, which was to be expected as the numbers of possible wrong answers are fewer. Images were anonymised three times and thus analysed in three different orders to avoid readers recalling images from previous rounds. This can be interpreted as the ^11^C-Choline PET/CT being very robust, where readers reproduce their own results reliably, experts and non-experts alike.

In a study of ^11^C-Choline PET/CT images from 21 patients and 4 observers, Noltes et al. reported a kappa value of 0.674, or good inter-observer variation when using a scan acquisition time similar to ours (i.e. 10 min) (Noltes et al. 2019). In a study of parathyroid imaging using ^99m^Tc sestamibi, Dalar et al. (Dalar et al. 2012) found ‘good’ to ‘very good’ inter- and intra-observer agreement, which is also in accordance with our findings.

The kappa values presented in this manuscript are slightly higher than those found by Noltes et al. Their readers were experienced (3–10 years) in nuclear medicine, but their level of experience with this particular type of imaging is unclear and may account for the slight difference (Noltes et al. 2019).

Although this study demonstrates a risk of slight bias favouring the expert, overall agreement was good when comparing the expert with the non-expert. This was true when determining which side (left or right) as well as the cranio-caudal location (upper left, lower left, upper right and lower right). The intra-observer kappa value for the expert reader was 0.90 and 0.87 for the two categories respectively, whereas the inter-observer kappa values, whilst still high, were slightly lower at 0.74–0.80. This does not differ from a frequently recognised pattern, where intra-individual variation is most often lower than the inter-individual variation. Thus, it is possible to analyse the ^11^C-Choline PET/CT images with high accuracy provided pre-existing experience with PET/CT-imaging. Nonetheless, there is still something to be gained by experience.

High sensitivities and near perfect specificities support the same impression: that non-experts can determine both HPGs and non-HPGs with high confidence as compared to the expert reader. However, a brief training period as well as continuous access to 11C-Choline PET/CT images are necessary in order to obtain a maintain expertise. As ^11^C-Choline has a short half-life of only 20 min, ^11^C-Choline PET/CT scans can only be performed at centres that have access to a cyclotron laboratory, limiting the method to larger centres.

^18^F-Choline has a longer half-life and could more easily be disseminated to smaller centres without access to a cyclotron laboratory. However, whether or not the two tracers are readily interchangeable has yet to be thoroughly investigated, as is the inter- and intra-observer agreement of ^18^F-Choline PET/CT.

## Conclusions

In this double-blinded study of inter- and intra-observer agreement of ^11^C-Choline PET/CT for location of HGPs, we conclude that an expert reader can replicate own readings with a near perfect degree of confidence, whilst the (nuclear medicine specialist) non-experts replicated the expert’s results with a high degree of confidence with little or no experience in parathyroid ^11^C-Choline PET/CT imaging. Based on these findings, we suggest that non-expert readers can analyse these images after only a brief training period of approximately 40 PET/CT images. Hence, the method may be readily disseminated to sites with a moderate number (i.e. 100-150 yearly cases corresponding to the number of images analysed in this paper) of cases with no concern for decreased quality in analysis, though a brief training period and access to a cyclotron laboratory are prerequisites.

## Supplementary Information

**Additional file 1: Table S1.** Number and location of HPGs as described by each reader. **Figure S1.** Agreement plots for intra-observer agreement on anatomical location including none-none agreement. Legend: From the expert, non-expert 1 and non-expert 2 readers. UR: upper right, LR: Lower right, UL: upper left, LL: lower left. In this figure we included cases where both readers find no HPG (in text referred to as “none-none-agreement”. **Figure S2.** Agreement plots for intra-observer agreement on anatomical location including none-none agreement. Legend: From the expert, non-expert 1 and non-expert 2 readers.

## Data Availability

The datasets used and/or analysed during the current study are available from the corresponding author on reasonable request.

## Abbreviations

HPG: Hyperfunctioning parathyroid gland
PET/CT: Positron emission tomography/computed tomography
PHPT: Primary hyperparathyroidism
PTx: Parathyroidectomy
SPECT/CT: Single-photon emission computed tomography/computed tomography
