# Supplementary material for: Locating hyperfunctioning parathyroid glands using 11C-Choline PET/CT: an inter- and intra-observer variation study
Source: Eur J Hybrid Imaging. 2021 Jul 6;5:13. doi: 10.1186/s41824-021-00108-z (PMC8257818; doi:10.1186/s41824-021-00108-z)
Supplement: Supplementary file 1 — Additional file 1: Table S1. Number and location of HPGs as described by each reader. Figure S1. Agreement plots for intra-observer agreement on anatomical location including none-none agreement. Legend: From the expert, non-expert 1 and non-expert 2 readers. UR: upper right, LR: Lower right, UL: upper left, LL: lower left. In this figure we included cases where both readers find no HPG (in text referred to as “none-none-agreement”. Figure S2. Agreement plots for intra-observer agreement on anatomical location including none-none agreement. Legend: From the expert, non-expert 1 and non-expert 2 readers. [file 41824_2021_108_MOESM1_ESM.docx]

**Locating hyperfunctioning parathyroid glands using 11C-Choline PET/CT :**

**An inter- and intra-observer variation study**

**-**

**SUPPLEMENTARY INFORMATION**

**Table S1** Number and location of HPGs as described by each reader

|  |  | **Expert reader** | | | **Non-expert 1** | | | **Non-expert 2** | | |
| --- | --- | --- | --- | --- | --- | --- | --- | --- | --- | --- |
| Round |  | 1 | 2 | 3 | 1 | 2 | 3 | 1 | 2 | 3 |
| Number of HPGs | 0 | 1 | 0 | 0 | 7 | 4 | 4 | 0 | 0 | 0 |
|  | 1 | 36 | 37 | 35 | 31 | 32 | 33 | 39 | 39 | 38 |
|  | 2 | 3 | 3 | 5 | 2 | 4 | 3 | 1 | 1 | 1 |
| Location^1^ | Right | 15 | 16 | 15 | 12 | 12 | 13 | 17 | 16 | 15 |
|  | Left | 21 | 22 | 21 | 19 | 21 | 21 | 22 | 23 | 23 |
|  | Both | 3 | 2 | 4 | 2 | 3 | 2 | 1 | 1 | 1 |
| Location^2^ | UR | 5 | 6 | 6 | 3 | 4 | 4 | 5 | 5 | 4 |
|  | LR | 13 | 12 | 14 | 11 | 11 | 11 | 13 | 12 | 12 |
|  | UL | 6 | 7 | 6 | 7 | 8 | 8 | 6 | 8 | 6 |
|  | LL | 18 | 18 | 19 | 14 | 17 | 16 | 17 | 16 | 18 |
| Certainty | Low | 3 | 1 | 5 | 4 | 5 | 2 | 6 | 4 | 2 |
|  | Moderate | 5 | 4 | 2 | 8 | 7 | 9 | 7 | 10 | 7 |
|  | High | 32 | 35 | 33 | 28 | 28 | 28 | 27 | 26 | 30 |
|  | Average* | 2.7 | 2.9 | 2.7 | 2,6 | 2,6 | 2,7 | 2,5 | 2,6 | 2.7 |
| ^1^ Location relative to the thyroid gland, Right, left or both  ^2^ Location relative to the thyroid gland, UR = upper right, LR = lower right, UL = upper left, LL = lower left  * Calculated as the average of certainties, when low = 1, moderate = 2, and high = 3 | | | | | | | | | | |

**Figure S1** Intra-observer agreement plots for each reader individually. Including none-none-agreement


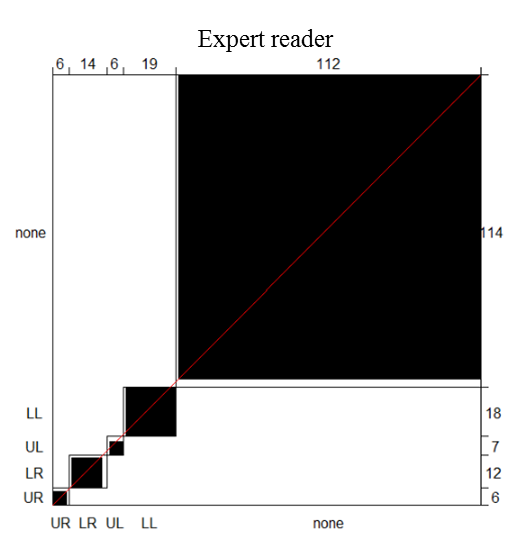

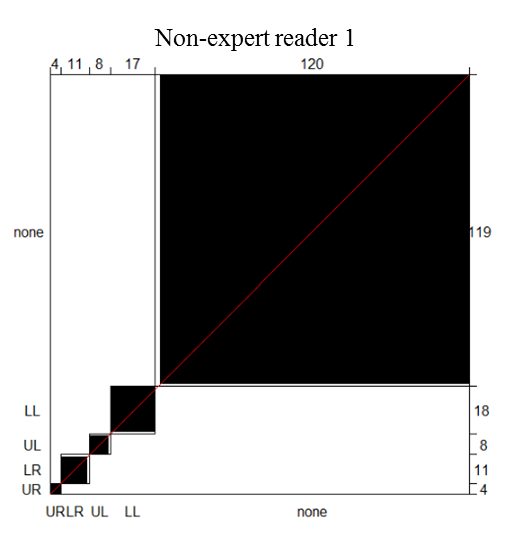


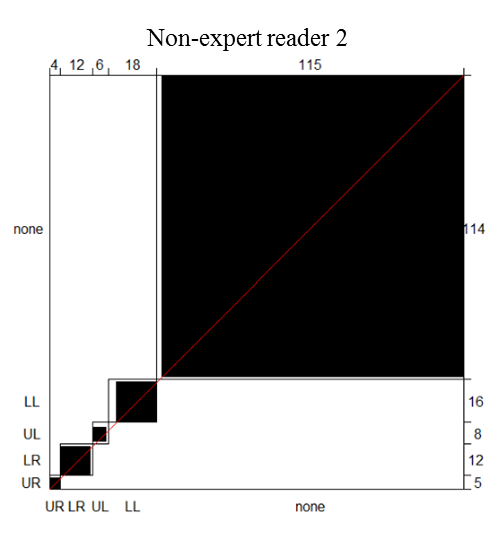


UR: upper right, LR: Lower right, UL: upper left, LL: lower left.

“None-none-agreement” referrers to cases where both readers find no HPG

**Figure S2** Inter-observer agreement plots between the expert and the two non-experts individually. Including none-none-agreement


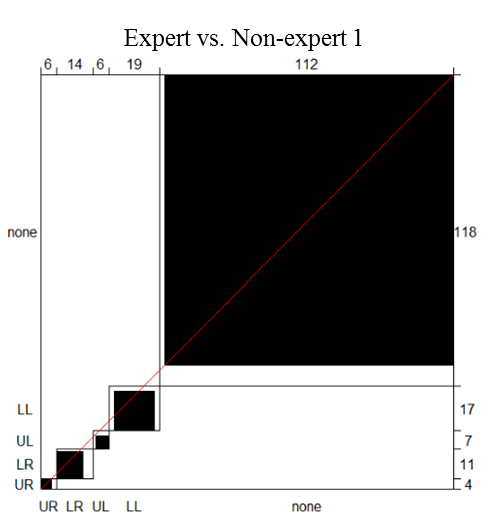

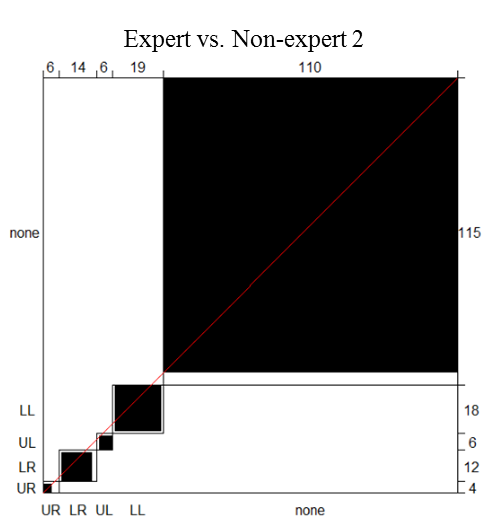


UR: upper right, LR: Lower right, UL: upper left, LL: lower left.

“None-none-agreement” referrers to cases where both readers find no HPG
